# Assessment of megabase-scale somatic copy number variation using single-cell sequencing

**DOI:** 10.1101/gr.198937.115

**Published:** 2016-03

**Authors:** Kristin A. Knouse, Jie Wu, Angelika Amon

**Affiliations:** 1 Koch Institute for Integrative Cancer Research, Department of Biology, Howard Hughes Medical Institute, Massachusetts Institute of Technology, Cambridge, Massachusetts 02139, USA;; 2 Division of Health Sciences and Technology, Harvard Medical School, Boston, Massachusetts 02115, USA;; 3 The Barbara K. Ostrom (1978) Bioinformatics and Computing Facility in the Swanson Biotechnology Center, Koch Institute for Integrative Cancer Research, Massachusetts Institute of Technology, Cambridge, Massachusetts 02139, USA

## Abstract

Megabase-scale copy number variants (CNVs) can have profound phenotypic consequences. Germline CNVs of this magnitude are associated with disease and experience negative selection. However, it is unknown whether organismal function requires that every cell maintain a balanced genome. It is possible that large somatic CNVs are tolerated or even positively selected. Single-cell sequencing is a useful tool for assessing somatic genomic heterogeneity, but its performance in CNV detection has not been rigorously tested. Here, we develop an approach that allows for reliable detection of megabase-scale CNVs in single somatic cells. We discover large CNVs in 8%–9% of cells across tissues and identify two recurrent CNVs. We conclude that large CNVs can be tolerated in subpopulations of cells, and particular CNVs are relatively prevalent within and across individuals.

Copy number variants (CNVs) can range in size from hundreds to millions of base pairs. Copy number changes affect approximately seven times as many base pairs as single-nucleotide variants and are major contributors to inter-individual differences (Sudmant et al. 2015). More than 65% of individuals harbor a germline CNV of at least 100 kb, and at least 1% of individuals have a CNV exceeding 1 Mb (Itsara et al. 2009). Although megabase-scale CNVs could be considered collectively common, the specific CNVs themselves are rare and often associated with disease (Girirajan et al. 2011). Not surprisingly, large CNVs experience negative selection, and their existence in a population is largely due to de novo events (Itsara et al. 2010).

Although germline, megabase-scale CNVs are found in 1% of individuals, the prevalence of somatic CNVs is only beginning to be investigated. Array-based analyses of populations of cells from many individuals provided initial insight into this question. These studies identified megabase-scale somatic aberrations in up to 4% of individuals; however, the sensitivity was limited to CNVs present in >5% of cells (Forsberg et al. 2012; Jacobs et al. 2012; Laurie et al. 2012). These studies are thus blind to alterations that arise late in development or adversely affect fitness, as this would limit their propagation in a cell population. With the emergence of methods to amplify the genome of a single cell, single-cell sequencing now provides an alternate means of assessing the prevalence of somatic CNVs and offers the advantage of detecting variants that exist in as few as one cell. Recently, two groups performed low-coverage sequencing of single human neurons and reported at least one megabase-scale CNV in >40% of neurons (McConnell et al. 2013; Cai et al. 2014). These findings suggest much greater tolerance of large somatic CNVs compared to germline CNVs and raise the interesting possibility that somatic genomic heterogeneity contributes to phenotypic diversity within a tissue. However, it is still unclear how CNV detection methods perform when applied to individual cells, as single-cell sequencing poses unique problems for CNV detection. First, single cells are usually sequenced at very low coverage. Second, genome representation in the sequencing library can vary independently of copy number due to inefficient and uneven genome fragmentation and amplification. Moreover, any alterations identified in a single cell cannot be verified by an independent method. Therefore, it is imperative that appropriate quality control and analytic methods are used such that the sensitivity (the likelihood that a real CNV of defined size is detected) and specificity (the likelihood that a detected CNV represents a real change in copy number) of an approach are known and optimized in the context of single-cell sequencing data.

Here, we use a variety of methods to quantify the sensitivity and specificity of different approaches for megabase-scale CNV detection in single-cell sequencing data. We develop an approach with higher specificity than those used previously and use this approach to analyze single-cell sequencing data from normal human brain and skin. From this analysis, we infer the prevalence of megabase-scale CNVs across somatic tissues.

## Results

### Characterizing sequencing data from single somatic cells

We previously isolated single cells from fresh postmortem brain and skin samples from four adults without neurologic or dermatologic disease (Knouse et al. 2014). Genomic DNA from a total of 105 brain cells (∼75% of which are neurons) from all four individuals and a total of 55 keratinocytes from two of these individuals were amplified by linker adapter PCR and sequenced at low coverage (0.1×) (Supplemental Table 1).

To quantify variation in read depth across the genome and identify cells suitable for analysis, we previously calculated a variability score (VS) for each cell (Knouse et al. 2014). The variability score is generated by averaging the standard deviation in read depth in sliding windows across each chromosome and averaging the average standard deviation for the three autosomes with highest variability. Although this is suitable for whole-chromosome copy number analysis, it could bias subchromosome copy number assessment as copy number changes within each chromosome could increase the VS. To assess the impact of CNVs on VS, we recalculated the VS of each cell by excluding windows with read depths above or below threshold for diploid copy number. The VS of only three of 160 cells changed when we excluded nondiploid regions of the genome. In these three cells, the VS changed by <0.02 (Supplemental Fig. 1A). This analysis indicates that copy number changes are not responsible for the majority of read depth variation. Regardless, we used the recalculated VS for all subsequent analyses.

In our data set, the variability scores (VSs) ranged from 0.14 to 1.03, with a median of 0.19 and a long upper tail (Supplemental Fig. 1B). The majority of cells with high VS were brain cells, and the median VS in brain was significantly higher than in skin (0.2 versus 0.18, Mann-Whitney *U* test, *P* < 0.02). Cells with high VSs were characterized by a wide spread of read depths across adjacent genomic windows (Supplemental Fig. 1C, middle panel) and/or an abundance of segments with copy number changes, many of which were non-integer and therefore incompatible with gains and losses in a single cell (Supplemental Fig. 1C, bottom panel, blue boxes). The VS is therefore an unbiased measure of sequencing data quality and a valid criterion for inclusion or exclusion of a cell from copy number analysis.

### Optimizing sensitivity and specificity of CNV detection in single-cell sequencing data

To determine the prevalence of CNVs in brain and skin, we tested two methods for copy number analysis. Both approaches quantify sequencing reads in genomic windows of ∼500 kb and adjust read counts for mappability and GC bias within each window. The two approaches differ in how copy number segments are inferred from read depth in each window. One uses a hidden Markov model (HMM) (Ha et al. 2012), whereas the other uses circular binary segmentation (CBS) (Olshen et al. 2004). Both HMM and CBS feature an adjustable parameter that determines the likelihood that a change in copy number is identified. For HMM, the adjustable parameter E dictates the probability of extending a segment of defined copy number. Thus, as E is lowered, more copy number changes are called. For CBS, the adjustable parameter α dictates the significance level required to accept a change in copy number. Therefore, higher levels of α allow for more copy number changes. Changing the values of these two parameters therefore adjusts the sensitivity and specificity of the two approaches.

To broadly test the performance of these two algorithms, we simulated gains and losses ranging from 2.5 to 20 Mb in sequencing data from single cells with different variability scores. For each cell, we determined the number of simulated and nonsimulated CNVs that were detected by HMM and CBS at different values of E and α. Several trends emerged. First, HMM had better sensitivity for gains, and CBS had better sensitivity for losses (Supplemental Fig. 1D, first and third panels). Second, overall sensitivity could be increased by reducing E or increasing α, improving the detection of 2.5 and 5 Mb CNVs. Notably, doing so also resulted in the detection of many nonsimulated CNVs, especially in cells with high VS (Supplemental Fig. 1D, second and fourth panels). Finally, for any algorithm at any parameter value, in cells with VS > 0.3 there was a dramatic reduction in the detection of simulated CNVs, especially gains, while as many as 100 nonsimulated CNVs were identified (Supplemental Fig. 1D).

**Figure 1.**
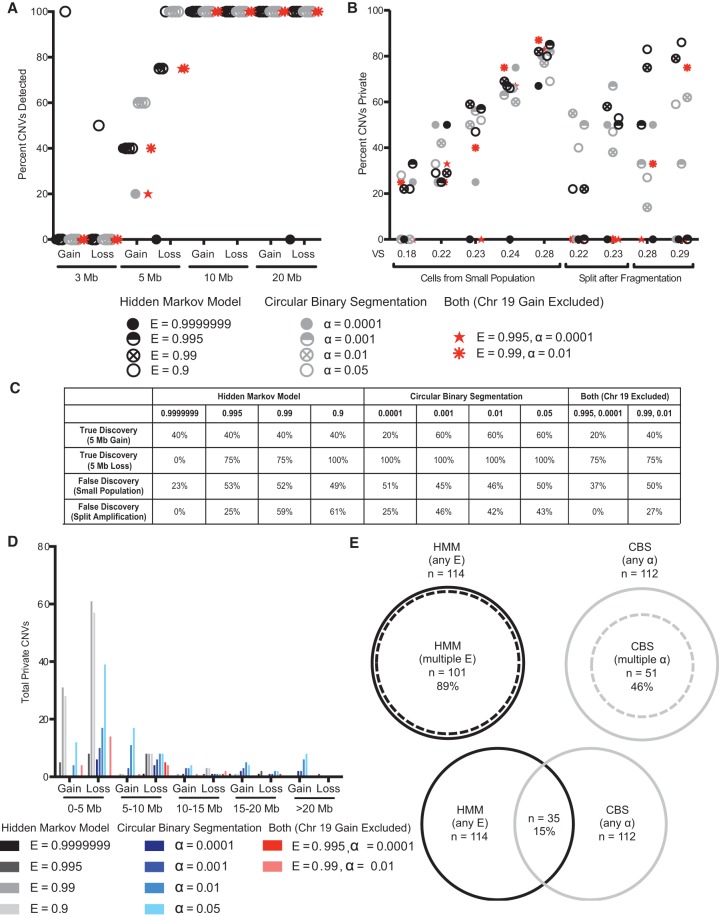
Testing sensitivity and specificity of CNV detection in single-cell sequencing data. (*A*) The discovery rate of known CNVs ranging from 3 to 20 Mb in single cells using HMM, CBS, or both at different values of E or α. Four or five cells were sequenced for each CNV. (*B*) The average proportion of CNVs that are private in individual cells sharing a recent ancestor or DNA split after fragmentation from a single cell when analyzed by HMM, CBS, or both at various parameters. (*C*) The true discovery rates (for 5-Mb gains and losses) and the false discovery rates (averaged across samples from small population or split amplification) for CNV detection using HMM, CBS, or both at different values of E or α, regardless of VS. (*D*) The distribution of private CNV type and size in individual cells sharing a recent ancestor or DNA split after fragmentation from the same cell when analyzed by HMM, CBS, or both at various parameters. (*E*) The proportion of private CNVs called by more than one parameter of HMM or CBS and the proportion of private CNVs called by both HMM and CBS.

To test the sensitivity of these approaches to real CNVs, we sequenced single fibroblasts from individuals known to harbor germline CNVs of defined size. All methods except HMM at E = 0.9999999 reliably detected 10 and 20 Mb gains and losses, whereas only HMM at E = 0.9 detected CNVs <5 Mb (Fig. 1A). We conclude that CNVs 5 Mb or larger can be detected by multiple approaches in single cells sequenced at 0.1× coverage.

Our simulations showed that algorithm sensitivity could be adjusted to identify CNVs of <5 Mb. However, this led to increased detection of nonsimulated CNVs, suggesting that specificity was compromised (Supplemental Fig. 1D, second and fourth panels). In these simulation experiments, as with all single-cell sequencing experiments, it is impossible to determine whether the nonsimulated CNVs represent true CNVs undetectable at less sensitive parameters or false CNVs caused by random fluctuations in read depth that are inappropriately identified as CNVs when sensitivity is increased. The next best way to verify CNVs is to sequence cells that are closely related to one another, ideally the two products of a cell division. Barring errors during DNA replication, two daughter cells should have identical or complimentary CNVs. A CNV present in only one of the cells, henceforth called a private CNV, therefore likely represents a false positive CNV or a CNV that failed to be detected in the other cell(s).

We expanded a single fibroblast in culture for approximately seven divisions to yield approximately 100 cells and sequenced five cells from this population. As any two cells in this population have been genetically distinct for at most seven generations, most CNVs should be shared by multiple cells. As a complimentary approach, we isolated single cells and split the cell contents in half after cell lysis and DNA fragmentation but prior to whole-genome amplification. As the lysis and fragmentation steps generate DNA fragments ∼1000-fold smaller than the windows at which we bin reads, the CNVs should be identical between the two samples. For both of these experiments, we then calculated the proportion of CNVs that were private in each cell or sample.

We were surprised to find that for most parameters of HMM and CBS, over half of the identified CNVs were private (Fig. 1B). Most of these private CNVs likely represent false CNVs, rather than real CNVs that failed to be detected in related cells or samples, because only 17% of private CNVs identified for a given algorithm at a less sensitive parameter were subsequently identified in other cells by the same algorithm using a more sensitive parameter. Across all algorithms and parameters, the private CNVs ranged in length from 0.5 to 34 Mb with the majority of private CNVs (63%) <5 Mb (Fig. 1D). Not surprisingly, the least sensitive algorithm and parameter, HMM at E = 0.9999999, was the most specific, with seven of nine cells lacking private CNVs when analyzed by this method. We also noted, especially in the split reactions, that increasing HMM and CBS sensitivity led to reduced specificity (Fig. 1B). Importantly, although in a given sample HMM or CBS tended to identify the same private CNV at varying levels of sensitivity, only 15% of private CNVs were identified by both HMM and CBS (Fig. 1E). All the private CNVs identified by both HMM and CBS were unique to a given cell with the exception of a 20 Mb gain on Chromosome 19, which was identified in four of the nine samples. This CNV was not detected when cells were sequenced at higher (1×) coverage, indicating that it is an artifact of low-coverage sequencing.

From these experiments, we conclude that private CNVs, the majority of which we believe to be false CNVs, can occur at the scale of megabases and can account for around half of all putative CNVs when algorithm parameters are adjusted to increase the sensitivity to detect CNVs 5 Mb and smaller. Furthermore, we find that HMM and CBS identify different private CNVs, suggesting that using both algorithms simultaneously could enhance specificity. Indeed, when we only considered CNVs detected by both HMM at E = 0.995 and CBS at α = 0.0001 and ignored the common gain on Chromosome 19, the prevalence of private CNVs fell to zero in two of the five cells from a small population and all of the split samples (Fig. 1B). Importantly, doing so did not compromise the sensitivity for CNVs exceeding 5 Mb (Fig. 1A). We decided that the overlap of HMM and CBS at E = 0.995 and α = 0.0001, respectively, afforded the best combination of sensitivity and specificity for the purpose of detecting megabase-scale CNVs in somatic cells. These parameters allow for the detection of all gains and losses 10 Mb and larger. Approximately half of 5-Mb CNVs can also be detected, with losses more easily detected than gains (Fig. 1C).

### Prevalence of somatic CNVs

**Figure 2.**
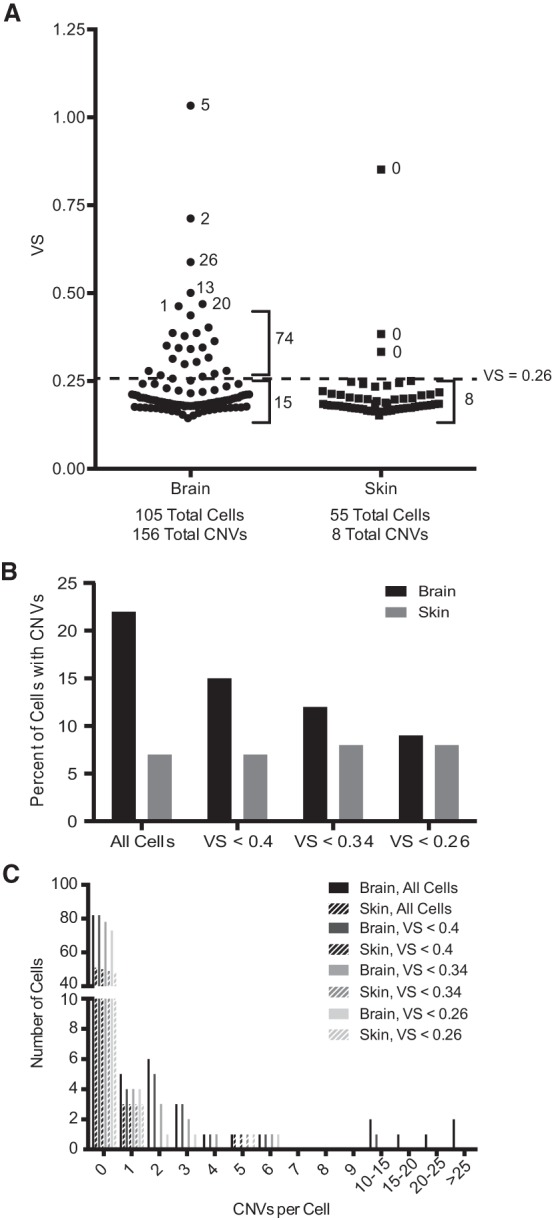
Relationship between variability score and CNV prevalence. (*A*) The variability scores for all brain and skin cells sequenced. The number adjacent to a given point or group of points indicates the total number of CNVs identified in that cell or group of cells. The dashed line indicates a VS cutoff of 0.26. (*B*) The percentage of brain and skin cells harboring at least one megabase-scale CNV using different VS cutoffs to exclude cells from analysis. (*C*) The distribution of the total number of megabase-scale CNVs per cell in brain and skin using different VS cutoffs to exclude cells from analysis.

We applied the combination of HMM and CBS at E = 0.995 and α = 0.0001, respectively, to all brain and skin cells. We ignored the gain on the proximal portion of Chromosome 19 that we found to be an artifact of low sequencing coverage and only considered CNVs present on autosomes. In brain, but not skin, we observed a positive correlation between the VS of a cell and the number of CNVs identified (Pearson *r* = 0.53, *P* < 0.0001) (Supplemental Fig. 2). Brain cells in the top tenth percentile for VS contained 72% of all CNVs identified in brain (Fig. 2A). Thus, the inferred CNV prevalence is strongly influenced by the cutoff used to select cells for analysis (Fig. 2B,C).

Understanding the causality between VS and CNVs is crucial given the positive correlation between a cell's VS and the number of CNVs in brain. High variability in read depth could lead to identification of CNVs that are not real. However, it is also possible that many CNVs increase read depth variability. Several observations argue against the latter. For one, when we recalculated VSs by excluding CNVs, the VSs hardly changed, indicating that the many CNVs in cells with high VS are not solely responsible for the high VS. Indeed, much of the variation in read depth in cells with high VS came in the form of fluctuations within the range of diploid copy number (Supplemental Fig. 1C, blue boxes). Furthermore, our data set includes a brain cell with six CNVs and a VS of only 0.22 (Supplemental Fig. 2, arrow), indicating that high VS is not an obligatory consequence of harboring many CNVs.

**Figure 3.**
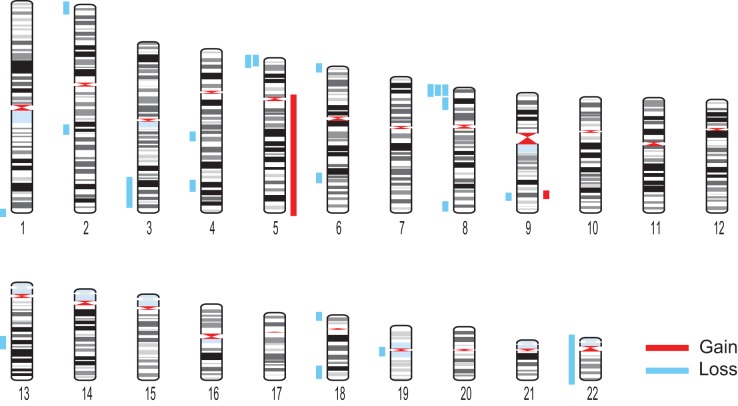
Megabase-scale CNVs in somatic cells. Karyogram showing the 23 gains and losses identified across 80 brain cells and 52 skin cells with VS <0.26. Gains and losses are represented by red and blue bars, respectively.

The preceding observations suggest that high VS is secondary to reasons other than variation in copy number, and many CNVs identified in such cells are likely to be false. Our private CNV analysis supports this hypothesis. The number of private CNVs was much higher in cells with high VS (Fig. 1B). Moreover, our simulations indicated that the sensitivity of CNV detection drops as VS exceeds 0.3 (Supplemental Fig. 1D, first and third panels). Because high VS not only complicates detection of real CNVs but also increases the discovery of false CNVs, we only considered cells with VS of <0.26 for our analysis (Fig. 2A, dashed line). This VS value renders the brain and skin cells equivalent in VS distribution (mean = 0.19 for both tissues). When applying HMM and CBS at E = 0.995 and α = 0.0001, respectively, to cells with VS < 0.26, we expect to detect 20% of 5-Mb gains, 75% of 5-Mb losses, and all CNVs 10 Mb and larger with a false discovery rate of <17% (Fig. 1). Of 132 brain and skin cells with VS < 0.26, we identified a total of 23 CNVs distributed across seven brain and four skin cells (Fig. 3; Supplemental Table 2).

### Validation of somatic CNVs

Although the nature of single-cell sequencing renders it impossible to validate the CNVs identified in a single cell by an orthogonal method, we used multiple analyses to increase our confidence that the CNVs we identified represent true gains and losses of genomic material. First, we resequenced four cells identified to have 6, 5, 3, and 1 CNVs and two cells with no CNVs at 10-fold higher coverage (1×). This analysis revealed that our CNV identification parameters are robust. The two cells without CNVs did not have any CNVs upon increased coverage. Furthermore, 87% of CNVs identified in cells at 0.1× coverage were also identified in cells at 1× coverage (Supplemental Table 3). Two small (<5.5 Mb) CNVs identified at 0.1× coverage were no longer detected at 1× coverage, and four small (<3 Mb) CNVs were identified at 1× coverage that were not detected at 0.1× coverage (Supplemental Table 3).

Resequencing cells at higher coverage allowed us to look for loss of heterozygosity (LOH) in putative deletions. We pooled all cells sequenced from each individual to identify single-nucleotide polymorphisms (SNPs) for which the individual was heterozygous. We then determined the state of these heterozygous SNPs in the cells sequenced at higher coverage. We first measured the distance between two successive heterozygous SNPs outside the regions of putative deletion in the single cells. These distances ranged from 2 bp to 58 Mb with a median of 2.8 Mb (Supplemental Fig. 3A). We then calculated the distance between heterozygous SNPs flanking (and, if present, within) putative deletions. The distance between heterozygous SNPs flanking/inside putative deletions was significantly higher than the distance between heterozygous SNPs outside putative deletions for two of the three cells (Mann-Whitney *U* test, *P* = 0.014, *P* < 0.0001) (Supplemental Fig. 3A). Encouraged by this result, we analyzed the state of SNPs within the putative deletions. At 1× coverage, the status of only a handful of SNPs could be assessed in each putative deletion. However, when we combined all SNPs for the 13 putative deletions that could be analyzed, there was a significant depletion of heterozygous SNPs within the putative deletions compared to outside the alleged deletions (Fisher's exact test, *P* = 0.0001). Nine of the 13 putative deletions did not harbor a single heterozygous SNP (Supplemental Fig. 3B). The remaining four deletions had a single heterozygous SNP. For one of these deletions, a loss on the proximal portion of Chromosome 8, the heterozygous SNP occurred within the first 500 kb of the putative deletion and thus could be a consequence of binning reads in ∼500 kb windows. For two other deletions, the heterozygous SNP was within at least 1.5 Mb of one boundary. This suggests that the CNV either was identified incorrectly or that there are two deletions joined by a small region of normal copy number. Finally, one heterozygous SNP was identified in a deletion that was not detected upon sequencing at higher depth, further supporting the conclusion that this deletion is not real.

We also analyzed 13 putative deletions in two cells with VS > 0.26. As before, we observed a significant increase in the distance between heterozygous SNPs within deletions as compared to outside deletions for both cells (Mann-Whitney *U* test, *P* = 0.0013, *P* < 0.0001) (Supplemental Fig. 3C). However, of the 13 deletions, only three lacked heterozygous SNPs, and there was no significant depletion of heterozygous SNPs within the putative deletions compared to outside these deletions (Fisher's exact test, *P* = 0.892) (Supplemental Fig. 3D). Together, our SNP analysis revealed that in low-coverage, single-cell sequencing data, continuous homozygosity can span megabases even in allegedly euploid regions. Thus, loss of heterozygous SNPs cannot rigorously confirm megabase-scale deletions, but the identification of heterozygous SNPs can identify false deletions. The significant increase in the distance between heterozygous SNPs flanking/inside deletions combined with the significant decrease in heterozygous SNPs within deletions in our data set supports but does not prove their existence. However, the identification of heterozygous SNPs within many of the deletions from cells excluded from analysis underscores the association between high VS and false CNVs and justifies the exclusion of such cells from analysis.

Although loss of heterozygosity is consistent with a deletion, it cannot distinguish a true genomic deletion from loss of DNA during cell lysis or whole-genome amplification. However, in contrast to loss of DNA during whole-genome amplification, most mechanisms that would generate true genomic deletions would join two previously separated regions of the genome. This juxtaposition could be identified by paired-end sequencing, assuming the fragment containing the junction did not drop out during whole-genome amplification. To determine whether we could identify such chimeric DNA fragments, we resequenced one cell harboring multiple deletions to 2× coverage with paired-end reads. We identified multiple (3–5) discordant reads flanking the breakpoints for half the deletions. However, we also identified thousands of discordant reads mapping to distant positions within and across chromosomes in allegedly euploid regions, and in some cases, a handful mapped to similar positions as they did for half of the putative deletions. We suspect this is due to chimera formation during whole-genome amplification. In light of the not insignificant probability that junction fragments drop out during whole-genome amplification (low signal), the high level of discordant reads in euploid regions (high background), and the financial costs associated with resequencing at higher coverage, we concluded that paired-end sequencing is not a viable approach for validating CNVs identified across many single cells. In summary, although SNP analysis can provide supporting evidence for the existence of deletions, it remains difficult to validate putative CNVs identified across many cells. This emphasizes the importance of using a CNV detection algorithm that minimizes the false discovery rate.

### Characteristics of somatic CNVs

**Figure 4.**
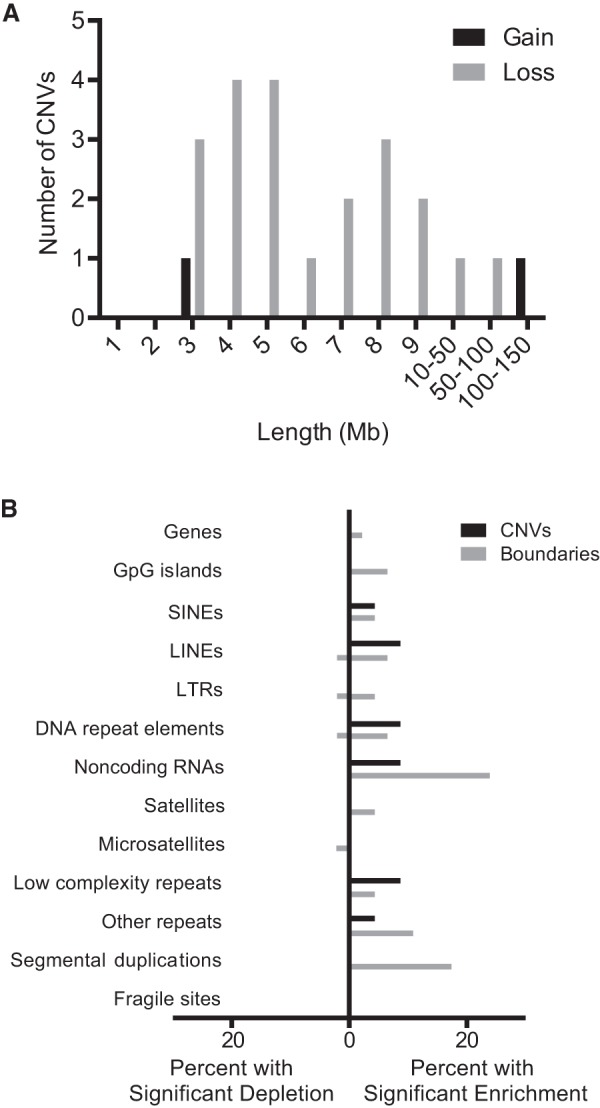
Characteristics of megabase-scale CNVs in somatic cells. (*A*) The distribution of CNV length among CNVs identified in brain and skin cells with VS <0.26. (*B*) The percentage of CNVs and CNV boundaries showing significant enrichment or depletion of various genomic features.

Using the CNVs detected by our empirically validated detection algorithm and exclusion criteria (Fig. 3; Supplemental Table 2), we went on to characterize the CNVs identified in brain and skin. To avoid biasing the data, we kept the two deletions that disappeared at higher sequencing depth in our data set and did not incorporate the four additional CNVs identified at higher depth. Our analysis revealed that 9% of brain cells and 8% of skin cells harbor at least one megabase-scale CNV (Fig. 2B). Across the individuals we analyzed, the prevalence of cells harboring megabase-scale CNVs ranged from 0% to 20% in brain and 4% to 11% in skin (Supplemental Fig. 4). Interestingly, CNV occurrence was not independent, as the 23 CNVs were distributed among only 11 of 132 cells (Poisson *P* < 10^−5^) (Fig. 2C). Losses were much more common than gains, but given that one of the gains comprised an entire chromosome arm, losses affected only slightly more of the genome (186.23 Mb of losses versus 137.4 Mb of gains) (Fig. 4A). Telomeric CNVs were highly enriched (Fisher's exact test, *P* < 0.0001), with 11 of the 23 CNVs extending to the telomere.

To determine whether CNVs or their boundaries shared certain characteristics, we independently tested whether each CNV and a 1-Mb region centered at each CNV boundary (or the first 0.5 Mb of a telomeric boundary) were enriched for various genomic features compared to a random genomic region of equivalent size. Thirty-five percent of CNVs were enriched for at least one type of repetitive sequence, such as SINEs, LINEs, DNA repeat elements (i.e., DNA transposons), and noncoding RNAs (i.e., tRNA, rRNA, snRNA, scRNA, and srpRNA) (Fig. 4B). CNV boundaries were even further enriched for repetitive sequence, with 54% of boundaries enriched for at least one of these elements. Although CNV boundaries often had repetitive elements, only two CNVs showed enrichment for the same type of repetitive sequence at both boundaries (noncoding RNA in both cases). Additionally, eight boundaries (17% of all boundaries) were enriched for segmental duplications, with seven of these boundaries occurring at telomeres. However, no CNVs were enriched for segmental duplications at both boundaries.

In light of the low CNV burden—we identified 23 CNVs that in total comprise only 324 Mb (∼10%) of the genome—we were surprised to find two CNVs that recurred with nearly identical coordinates in at least two cells. The first was an ∼7-Mb loss on the proximal portion of Chromosome 5 in two brain cells from two different individuals (Fig. 3; Supplemental Table 2). The second was an ∼5-Mb loss on the proximal portion of Chromosome 8 in two brain cells from the same individual and one skin cell from a different individual. This region of Chromosome 8 has previously been identified as a rearrangement hotspot because of an abundance of segmental duplications at chromosome coordinates 0, 1, 7, and 8 Mb that predispose this region to nonallelic homologous recombination (Bailey et al. 2002; Yu et al. 2010). A study of 1000 individuals with developmental defects revealed megabase-scale CNVs on the proximal portion of Chromosome 8 in 1% of patients (Yu et al. 2010). This region of Chromosome 8 has also been identified as a peak region of deletion across multiple tumor types and contains the tumor suppressor *CSMD1* (Ma et al. 2009; Midorikawa et al. 2009; Zack et al. 2013). In light of these preexisting data, it seems likely that CNVs in this region occur more frequently, and perhaps provide a selective advantage, at the somatic level.

## Discussion

Low coverage sequencing of single cells is emerging as a popular and powerful tool to assess genomic heterogeneity in health and disease. However, it has been unclear what types of variants can be detected and, more importantly, what the likelihood is that the detected variants are real. Through a combination of in silico and in vivo approaches, we assessed the sensitivity and specificity of a variety of analytical approaches for CNV detection in low coverage, single-cell sequencing data. We developed an approach that allows for the robust yet specific detection of CNVs exceeding 5 Mb. We applied this approach to single-cell sequencing data from brain and skin to provide, to our knowledge, the first assessment of somatic CNVs in multiple tissues at genome-wide, single-cell resolution. We find that ∼10% of somatic cells harbor at least one megabase-scale CNV regardless of tissue of origin.

Our analysis shows that the specificity of CNV detection is extremely compromised when algorithm parameters are adjusted to detect CNVs <5 Mb and when cells exhibiting high variability in read depth are analyzed. In these cases, false positive CNVs can account for more than half of CNVs detected. This can explain the much higher prevalence of CNVs reported for neurons in two recent single-cell sequencing studies. McConnell et al. (2013) sequenced 110 neurons at ∼0.1× coverage and identified megabase-scale CNVs in 45 (41%) of these cells. Their data set included 14 cells with VS > 0.26, and CNVs were detected using CBS with α = 0.001. When we reanalyzed their data using our VS cutoffs and overlapping algorithms, we identified megabase-scale CNVs in 17% of cells. Cai et al. (2014) sequenced 26 neurons at ∼0.08× coverage, 19 of which they analyzed, and 13 (68%) of these cells were reported to harbor megabase-scale CNVs. In their data set, 16 cells had VS >0.26, and CNVs were detected using CBS with α = 0.02. When we analyzed the 10 cells with VS < 0.26 by our approach, we identified CNVs in only one neuron. Thus, the differences between our results and those reported by McConnell et al. (2013) and Cai et al. (2014) can be attributed to differences in analytic methods rather than differences in the cell populations.

We acknowledge that the majority of cells we excluded with VS > 0.26 were from brain. Thus, it is possible that the brain has a subset of cells with high CNV burden that was excluded from our data set. However, we believe that including cells with high VS is more likely to generate artifacts than to report on true CNVs for several reasons. For one, our specificity experiments show that cells with higher VS have a greater proportion of false CNVs. Moreover, our analysis of SNPs in cells with high VS revealed that the majority of their putative deletions were false. Finally, we note that CNVs do not appear to be responsible for high VS, and much of the variability in these cells is in the form of non-integer copy number changes. It is unclear why high VSs are more common in brain than skin but could reflect biological differences in chromatin structure that affect the efficiency of whole-genome amplification. Until we better understand the biological and/or technical origin of high VS, we must assume that the high CNV burden in cells with high VS is an artifact.

Our approach generated a data set of high confidence, megabase-scale somatic CNVs, leading to several conclusions about somatic copy number variation. First, we observe 10-fold more losses than gains. However, because one gain was much larger than all other CNVs, losses and gains affected a similar amount of the genome. We note the increased frequency of losses compared to gains could be secondary to our approach having better sensitivity to losses for CNVs <10 Mb. Second, somatic CNVs are not distributed uniformly throughout the genome but instead tend to occur at telomeres. Many of the CNVs were enriched for repetitive sequence such as SINEs, LINEs, DNA repeat elements, and noncoding RNAs. Presumably CNVs affecting repetitive sequence are better tolerated than CNVs enriched for coding sequence.

More than 20% of germline CNVs are associated with segmental duplications, and it is believed that nonallelic homologous recombination among these segmental duplications or other repetitive sequences is a common source of germline duplications and deletions (Sharp et al. 2005; Redon et al. 2006). On the other hand, high-resolution analyses of cancer genomes point to nonhomologous end joining and alternative end joining as the primary source of somatic deletions in tumors (Yang et al. 2013). We observed enrichment for repetitive sequences and segmental duplications at the boundaries of somatic CNVs, suggesting that nonallelic homologous recombination might also underlie somatic structural variation in nontransformed cells. However, only two CNVs were enriched for the same class of repetitive sequence at both breakpoints and no CNVs were flanked by segmental duplication on both sides. With limited resolution at breakpoints and without obvious repetitive sequence flanking both boundaries of most CNVs, we are unable to implicate specific molecular mechanisms in generating somatic CNVs in healthy tissues.

We were surprised to find two recurrent CNVs, deletions on the proximal portions of Chromosomes 5 and 8, in two and three cells, respectively. The deletion on Chromosome 8 thus occurs in 2% of all cells analyzed and accounts for 13% of all the CNVs we detected. That this particular CNV has been previously identified in multiple population-based copy number analyses and was never identified as a private CNV in our study further increases our confidence that this loss represents a real, recurrent CNV as opposed to a single-cell sequencing artifact. Moreover, its presence at the individual level and in cancer suggests that it not only may be prone to arising because of local genome structure but may also provide a selective advantage for cells that harbor it.

We find that megabase-scale CNVs are 10 times more prevalent at the somatic level compared to the organismal level. This suggests that these aberrations arise more frequently in mitotic cells and/or, more likely, that these changes are better tolerated when they occur sporadically in tissues. However, like germline CNVs, somatic CNVs show an inverse relationship between CNV size and prevalence. In this current analysis, we observe whole-chromosome copy number changes in 0.8% of cells. Subchromosome CNVs exceeding 10 Mb are present in 1.5% of cells, and CNVs between 5 and 10 Mb are found in 3.8% of cells. Extrapolating to events beyond our detection limit of 5 Mb, we expect that >15% of cells harbor CNVs smaller than 5 Mb.

We present and validate an approach that allows for detection of megabase-scale CNVs with high specificity in low coverage single-cell sequencing data. With this approach, we now have the power to address various questions of genomic heterogeneity in health and disease. It remains to be determined whether somatic CNVs accumulate with age and whether cells harboring these changes will undergo further transformation or contribute to tissue dysfunction. Sequencing cells at much higher coverage, which will necessitate collaboration across multiple groups and financial sources, will enable the characterization of even smaller variants. Further technological development catered toward single-cell sequencing will also help to enhance the sensitivity and specificity at any coverage level.

## Methods

### Tissues, cell lines, and sequencing data

The tissue sources were described previously (Knouse et al. 2014). The following cell lines harboring known CNVs were obtained from the NIGMS Human Genetic Cell Repository at the Coriell Institute for Medical Research: GM10374, GM05401, GM08263, GM05875, GM06801, GM13330, GM08696, and GM03918. Data from McConnell et al. (2013), Cai et al. (2014), and Knouse et al. (2014) were downloaded from the National Center for Biotechnology Information Sequence Read Archive using accession numbers SRP030642, SRP051114, and SRP041670, respectively.

### Single-cell whole-genome amplification and sequencing

Single-cell isolation, whole-genome amplification, and sequencing were performed as previously described (Knouse et al. 2014) with the following modifications. To sequence cells from a small population, single fibroblasts from GM08696 were transferred to individual wells of a 96-well plate using a homemade microaspirator. The cells were cultured until approximately 100 cells were present in a single well, at which point the contents of the well were harvested by trypsinization, and individual cells were prepared for sequencing. To sequence two separate amplifications of a single cell, the solution containing a lysed and fragmented cell was split into two separate tubes of equal volume. Both tubes were then subjected to whole-genome amplification using a half volume of each subsequent reagent.

### Quality control

Variability scores (VSs) for all cells were calculated previously (Knouse et al. 2014). To recalculate VS with CNVs excluded, windows with read depth at log_2_ ratios above the threshold for gain or loss were eliminated from the calculation.

### CNV simulation

Sequencing data from 12 samples with different VSs were used as input. To simulate copy number loss, we randomly down-sampled half the reads in the desired CNV interval. To simulate copy number gain, all the reads in the desired CNV region were retained while one-third of the reads outside the window were removed. Functions implemented in SAMtools (version 0.1.19) (Li et al. 2009) and BEDTools (version 2.17.0) (Quinlan and Hall 2010) were used in the region sampling. The lengths of these simulated CNVs varied from 2.5 to 20 Mb and, for each length, five CNVs were simulated throughout the genome. These modified sequencing data were then analyzed for CNVs as described below. A CNV of defined size was considered detectable if it was identified in at least three of five cases in a single cell.

### CNV detection using HMM

Sequence reads were trimmed to 40 nucleotides and aligned to the major chromosomes of human (hg19) using BWA (version 0.6.1) (Li and Durbin 2009) with default options. HMMcopy (version 0.1.1) (Ha et al. 2012) was used to detect CNVs by estimating copy number in 500-kb bins controlling for mappability (downloaded from UCSC Genome Browser; http://hgdownload.cse.ucsc.edu/goldenPath/hg19/encodeDCC/wgEncodeMapability/) and GC content (calculated by HMMcopy gcCounter). The parameter E was varied from default (E = 0.9999999) to E = 0.995, 0.99, 0.95, and 0.9 for testing. Log_2_ cutoffs of 0.4 and −0.35 were used for gains and losses, respectively. These cutoffs were set just below the minimum log_2_ ratio at which known CNVs were observed.

### CNV detection using CBS

The mappability track (http://hgdownload.cse.ucsc.edu/goldenPath/hg19/encodeDCC/wgEncodeMapability/wgEncodeCrgMapabilityAlign40mer.bigWig) downloaded from UCSC Genome Browser was processed to define dynamic windows containing 500-kb uniquely mapped locations in each window as previously described (McConnell et al. 2013). The GC percentages were computed for these windows. The reads were first mapped by BWA (version 0.6.1), and uniquely mapped reads were kept. PCR duplicates were then removed using MarkDuplicates from Picard (version 1.94). Read counts in the dynamic windows were summarized by BEDTools (version 2.17.0) coverageBed (Quinlan and Hall 2010). Readcount in each window was normalized by the genome-wide median read count of all windows with similar GC percentage, as measured in 1% intervals, then multiplied by 2 (McConnell et al. 2013). Log_2_ ratios were then used as input for DNAcopy in R (Olshen et al. 2004). The parameter α in the DNAcopy package was varied (0.0001, 0.001, 0.01, and 0.05) for testing. Cutoffs of 1.32 and 0.6 were used for gains and losses, respectively.

### CNV detection using both algorithms

Sequencing data from each cell were independently processed by the two methods described above at the specified values of E and α. A CNV was called only in regions in which both algorithms identified a CNV of the same type (loss or gain). If the two algorithms identified the same CNV but the boundaries differed, the coordinates of overlap were set as the boundaries of the CNV.

### SNP analysis

SAMtools (version 0.1.19) mpileup and BCFtools (Li et al. 2009) were used to identify variants in the BAM alignment output. VCFtools (version 0.1.8a) vcf-annotate (Danecek et al. 2011) was then used to match these variants to dbSNP build 138 (Sherry et al. 2001). For pooled samples, a mapping quality score of 30 and a read depth of 4 were required to identify heterozygous SNPs. The DP4 tags in the VCF files were used to characterize the status of known SNPs that are not located in repetitive regions defined by RepeatMasker track downloaded from UCSC Genome Browser.

### Enrichment analysis

To test for telomere enrichment, BEDTools shuffle was used to identify 10 random regions of the genome that were the same length as each CNV in our data set. The location of these 230 coordinates was then compared to the location of the 23 CNVs. To test for enrichment of other genomic features, the following BED format annotation files were downloaded from UCSC Genome Browser: segmental duplications (Segmental Dups), repeats (RepeatMasker), CpG islands (cpgIslandExt), and genes (refGene). Fragile sites identified by a previous study (Fungtammasan et al. 2012) were extracted and lifted over to hg19. BEDTools was used to overlap the CNVs and their boundaries to these annotation files and count the number of features per million base pairs. These densities were compared with the background feature densities throughout the whole genome, and *Z*-scores for each were calculated. A CNV or boundary was considered significantly enriched or depleted for a given feature if the *Z*-score was greater than 1.96 or less than −1.96, respectively.

## Data access

The additional sequencing data generated in this study (cells harboring known CNVs, cells sharing a recent ancestor, DNA split after fragmentation from a single cell, and cells resequenced at higher coverage) have been submitted to the NCBI Sequence Read Archive (SRA; http://www.ncbi.nlm.nih.gov/sra/) under accession number SRP041670.
